# *AtDOF5.4/OBP4*, a DOF Transcription Factor Gene that Negatively Regulates Cell Cycle Progression and Cell Expansion in *Arabidopsis thaliana*

**DOI:** 10.1038/srep27705

**Published:** 2016-06-14

**Authors:** Peipei Xu, Haiying Chen, Lu Ying, Weiming Cai

**Affiliations:** 1 Institute of Plant Physiology and Ecology, Shanghai Institutes for Biological Sciences, Chinese Academy of Sciences, 300 Fenglin Rd, Shanghai 200032, China

## Abstract

In contrast to animals, plant development involves continuous organ formation, which requires strict regulation of cell proliferation. The core cell cycle machinery is conserved across plants and animals, but plants have developed new mechanisms that precisely regulate cell proliferation in response to internal and external stimuli. Here, we report that the DOF transcription factor *OBP4* negatively regulates cell proliferation and expansion. OBP4 is a nuclear protein. Constitutive and inducible overexpression of *OBP4* reduced the cell size and number, resulting in dwarf plants. Inducible overexpression of *OBP4* in *Arabidopsis* also promoted early endocycle onset and inhibited cell expansion, while inducible overexpression of *OBP4* fused to the VP16 activation domain in *Arabidopsis* delayed endocycle onset and promoted plant growth. Furthermore, gene expression analysis showed that cell cycle regulators and cell wall expansion factors were largely down-regulated in the *OBP4* overexpression lines. Short-term inducible analysis coupled with *in vivo* ChIP assays indicated that *OBP4* targets the *CyclinB1;1*, *CDKB1;1* and *XTH* genes. These results strongly suggest that *OBP4* is a negative regulator of cell cycle progression and cell growth. These findings increase our understanding of the transcriptional regulation of the cell cycle in plants.

In contrast to animals, plants continue to generate new organs throughout their life cycles. Plant growth and development are closely coordinated to achieve the plant’s final size and shape^49^. Plant morphogenesis relies on spatially and temporally coordinated cell proliferation and differentiation^44^. Cell proliferation is increase the number of cells through the cell growth and division producing daughter cells. The core cell cycle regulatory mechanisms are conserved in eukaryotes. The cell cycle consists of the replication phase and the mitotic phase, which are separated by G1 and G2, two gap phases^6^. Cyclin-dependent kinases (CDKs) and their phosphorylated target genes, which trigger the onset of DNA replication and mitosis at the post-transcriptional level, play a central role in cell cycle regulation. Cell differentiation converts dividing cells into non-dividing cells and also determines cell fate. In many cases, cell differentiation occurs simultaneously with the endocycle. During the endocycle, the cell replicates its genome without cell division, resulting in cells with more than 4C of DNA content^49^. Previous study has shown that many genes are involved in endocycle initiation. *CDKB1;1*, a key regulatory gene in the G2-M phase transition, has been shown to negatively regulate the onset of endocycle^4^. High expression level of *ICK/KRP1* may suppress the endocycle and inhibit G2-M phase transition^45^,^65^. RBR1 regulates the switch from proliferation to endocycles by targeting the *CCS52A1* and *CSS52A2* genes^33^.

Cell cycle progression is also controlled at the transcriptional level in response to developmental and environmental signals^15^. A number of MYB transcription factors^9^,^27^, TCP transcription factors^1^,^29^ and E2F transcription factors^23^,^34^ can affect cell proliferation in a spatial, temporal or quantitative manner. Previous research has shown that *TCP20* and *MYB59* bind to the promoter of *CYCB1;1* and modulate its expression in G2-M phase^29^,^38^. *TCP15* controls endocycle onset by directly targeting the *CYCA2;3* and *RBR* (RETINOBLASTOMA-RELATED) genes^31^. The AP2 transcription factor *ANT* (AINTEGUMENTA) gene can maintain the meristematic competence of cells by regulating *CYCD3;1* expression^37^. However, the mechanisms by which developmental signals interact with plant cell cycle progression remain unclear.

DNA binding with one finger (DOF) proteins are a group of plant-specific transcription factors. A conserved N-terminal DNA-binding domain is present in typical DOF proteins, and the N-terminal domain can bind to DNA sequences harbouring an AAAG core motif and interact with other proteins. A divergent transcription regulation domain is present at the C terminus, this divergent C terminus is used for transcriptional regulation^51^,^58^. In *Arabidopsis*, the DOF transcription factor family has 37 members that play diverse roles in plant development and in response to environmental stimuli. In a screen for proteins that interact with the bZIP transcription factor OCS element binding factor 4 (OBF4), the OBF binding protein 1 (OBP1) was the first to be reported. Furthermore, other DOF transcription family proteins, OBP2 and OBP3, were identified to have the ability to bind OBF4 and to enhance their binding to the OCS element in the downstream target genes^21^. *OBP1* is involved in the control of cell division by targeting the core cell cycle regulator *CYCB3;3* and the S phase-specific transcription factor *DOF2.3*^47^. *OBP2* plays a role in the regulation of indole glucosinolate metabolism^48^, and *OBP3* has been characterized as a novel component of light signalling^54^.

This study identifies the function of DOF transcription factor *OBP4/DOF5.4* in cell cycle regulation. *OBP4* controlled plant growth by regulating core cell cycle genes involved in the replication machinery and cell expansion regulators. Correspondingly, *OBP4* overexpression resulted in the early onset of endocycle progression. Based on the expression profile and genetic results, we propose that *OBP4* is a novel regulator of cell cycle progression and cell expansion.

## Materials and Methods

### General methods

*Arabidopsis* seeds were sown in soil and grown in a growth chamber with a 16-h day length provided by fluorescent light at 120 μmol m^−2^s^−1^ and a day:night temperature of 22 °C-18 °C and relative humidity of 60–75%. Agrobacterium tumefaciens strain GV3101 was used to transform *Arabidopsis* thaliana Col-0 (35S::*OBP4*, RNAi::*OBP4*, p*OBP4*::GUS). Chemicals were purchased from Roche (Basel, Switzerland) or Sigma (St. Louis, MO). We used NCBI (http://www.ncbi.nlm.nih.gov/) and TAIR (http://www.*arabidopsis*.org/) to analyze gene sequences. We used GraphPad primer 5 (GraphPad Software Inc., San Diego, CA) to make diagrams. For induction experiments, plants were sprayed with 20 μm estradiol solution or grown in MS medium containing 20 μm estradiol.

### Plasmid constructs

To construct the 35S::*OBP4* vector, polymerase chain reaction (PCR) was used to amplify the *OBP4* CDS using Col-0 leaf cDNA as the template. The *OBP4* cDNA was cloned into a modified pHB plant transformation vector via the PstI and SacI sites. To construct the pER8::*OBP4* and pER8::*OBP4*::HA vectors, *OBP4* cDNA or *OBP4* cDNA fused to an HA tag were cloned into the pER8 vector via the SpeI and XhoI sites. To construct the pER8::*OBP4*::VP16 vector, *OBP4* cDNA was fused to the VP16 domain and cloned into the pER8 vector via the SpeI and XhoI sites. To construct the RNAi::*OBP4* vector, the forward and reverse fragments of a 3′-UTR *OBP4* sequence that does not have similarity with the UTRs of the other genes were linked to the pCAMBIA1301-RNAi vector. To construct the Promoter::GUS fusion vector, an approximately 2.0 kb 5′-UTR upstream of the ATG start codon was fused to the *Escherichia coli* GUS reporter gene in the pCAMBIA1300 vector. The primer sequences are shown in Supplementary Table S1.

### RNA isolation and Quantitative real-time PCR

The roots, stems, leaves, flowers, and siliques of five-week-old plant were harvested for RNA isolation using TRIzol reagent (Invitrogen, Grand Island, NY). 2 μg RNA was digested with DNase and reverse-transcribed by Superscript III reverse transcriptase (Invitrogen) to generate first-strand cDNA in a 20 μl reaction volume. Quantitative RT-PCR was performed in 96-well plates with 1 μl of a 1:5 dilution of the first-strand cDNA reaction and in a 10 μl volume on a LightCycler 96 Sequence Detection System (Roche). Transcript levels were measured based on SYBR Green technology using SYBR Green reagent (Applied Biosystems Applera, Darmstadt, Germany) according to the manufacturer’s instructions. Data were analyzed using the ΔΔC_T_ method. The qPCR results were analyzed using LightCycler 96 analysis software 1.1 (Roche). The *Arabidopsis ACTIN2* gene was used as a control. Primer sequences are shown in Supplementary Table S2.

### Subcellular localization of *OBP4*

*OBP4* sequences were cloned into PBI121 vector to construct fusion plasmid by using primers containing BamH1 and Sal1 sites. PBI121 plasmid was used as a control. The fusion construct and the control plasmid were transformed into *Arabidopsis* and tobacco leaf epidermal cells. Transformed cells were observed under a OLYMPUS FV1000 microscope.

### Culture and synchronization of *Arabidopsis* suspension cell

Col *Arabidopsis* Seeds were surface-sterilized and vernalized. Callus formation was induced under continuous darkness at 23 °C by placing root explants excised from 2-week-old seedlings onto callus induction medium. The rapidly dividing callus was inoculated into MS medium containing 3% w/v sucrose, 0.5 mg/L 2,4-D, 0.05 mg/L kinetin to establish suspension cultures,, and incubated on a rotary shaker at 110 rpm. The method to achieve synchronization was as previous described^62^.

### Flow Cytometry of Cell Cycle Progression

WT, pER8::*OBP4* and pER8::*OBP4*::VP16 transgenic *Arabidopsis* seeds were sterilized with 0.15% mercuric chloride and germinated on a plate. After induction with estradiol, nuclears were isolated from *Arabidopsis* leaves at different time points^55^. The DNA content of individual transgenic cells was determined by flow cytometry. Each sample was prepared three times and subjected to Beckman Coulter MoFlo XDP. A total of 10,000 nuclei were measured per analysis.

### ChIP assays

An HA-coding sequence was fused in frame to the end of the *OBP4* gene, and the gene fusion was sub-cloned under the estradiol-inducible promoter in the pER8 vector. The expression construct was then transformed into Col-0 plants. Two-week-old pER8::OBP4::HA transgenic plants that were grown on MS agar plates that expressed OBP4::HA via induction by estradiol for a period were used to conduct ChIP experiments according to a previously described method using anti-HA polyclonal antibodies (Roche:118674231)^64^. The qRT-PCR primers used are listed in Supplemental Table 2.

### For scanning electron microscopy

The leaves and hypocotyls were cut and sputter-coated with gold and further visualized by using a Hitachi JEOL JSM-6360LV SEM (scanning electron microscope).

### Microscopy and GUS staining

After removal of chlorophyll using 100% ethanol, epidermal cells were observed under a Olympus light microscope (Tokyo, Japan) and the size of cells were analyzed by cell^P Software, about thirty cells were observed of each genotype. β-Glucuronidase activity was determined histochemically as described^20^ and ten lines of transgenic plants were observed.

## Results

### *OBP4* is a DOF transcription factor family protein

**Figure 1:**
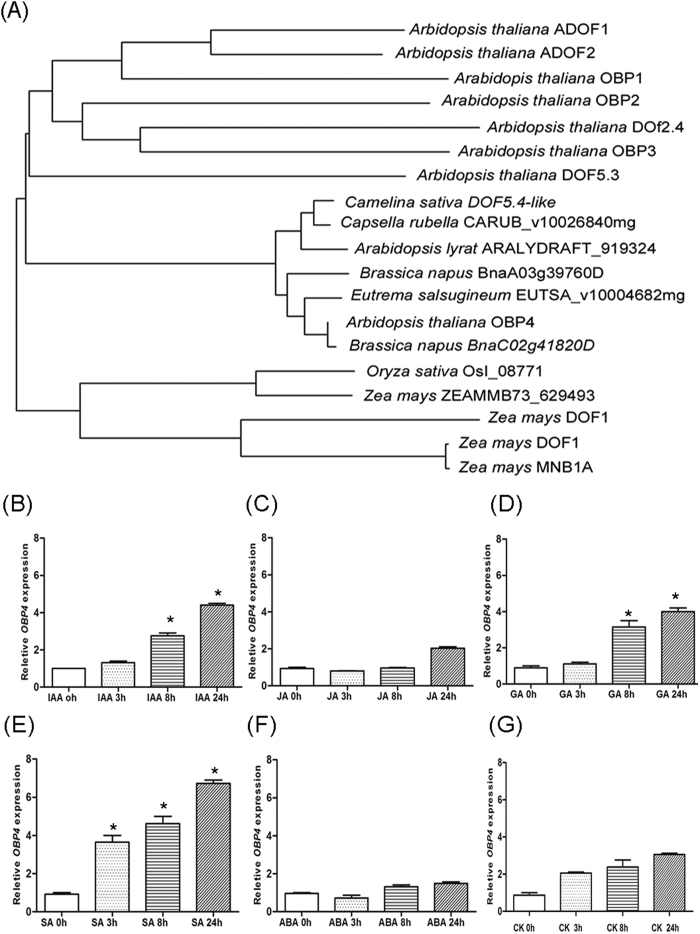
Homologous alignment and analysis of *OBP4* levels. (**A**) Phylogenetic comparison of DOF protein sequences from *Arabidopsis thaliana*, *Brassica napus*, *Oryza sativa, Capsella rubella*, *Camelina sativa* and *Eutrema salsugineum* in the database. Alignments were made by ClustalW2 and MEGA5.1 software using the default parameters and excluding positions with gaps. To determine *OBP4* expression in response to hormone treatment, eight-day-old seedlings were transferred to fresh medium (mock), or medium that contained (**B**) IAA (0.25 μM) (**C**) GA (0.25 μM) (**D**) JA (0.25 μM) (**E**) SA (0.5 μM) (**F**) ABA (0.1 μM) or (**G**) CK (0.1 μM) for the indicated period. RNA was then isolated from whole seedlings and analysed. Three biological and technical replicates were used. Asterisks indicate significant differences, p < 0.05.

The OBP1 protein was previously isolated^47^,^61^. Other members of the OBP family were identified using the sequence of the OBP1 DOF domain to screen an *Arabidopsis* cDNA library. *OBP4* is a previously uncharacterized, new member of the DOF family of proteins in *Arabidopsis*. The amino acid sequences of the OBPs (*OBP1*, *OBP2*, *OBP3*, and *OBP4*) are shown in Fig. S1. The OBP4 protein has 308 amino acids, and its DOF domain is located at the N terminus (boxed)^32^,^38^. The homology among the OBP proteins is restricted to the DOF domain. The specific characteristics of the OBPs are indicated in Fig. S1. In *OBP1* and *OBP2*, a serine-rich domain is observed, while in *OBP2*, an asparagine-rich domain is present. We further compared the OBPs to DOF proteins from other species in the database. We did not identify any significant homology between the OBP proteins and other DOF members other than the DOF domain. As shown in Fig. 1A, OBP4 is closely related to the *Brassica napus* BnaC02g41820D protein.

*OBP1* was first isolated through interactions with OBF proteins by an ocs element^61^. Ocs elements respond to salicylic acid (SA), and *OBP1-3* expression has been reported to be induced by SA. Furthermore, we assessed whether *OBP4* is affected by hormone treatment. After IAA and SA treatment, *OBP4* expression in the leaves increased substantially at 3 h and remained high at 24 h (Fig. 1B,E), while after 8 h of GA treatment, *OBP4* expression increased only slightly (Fig. 1D).

### *OBP4* gene expression and OBP4 protein localization

**Figure 2:**
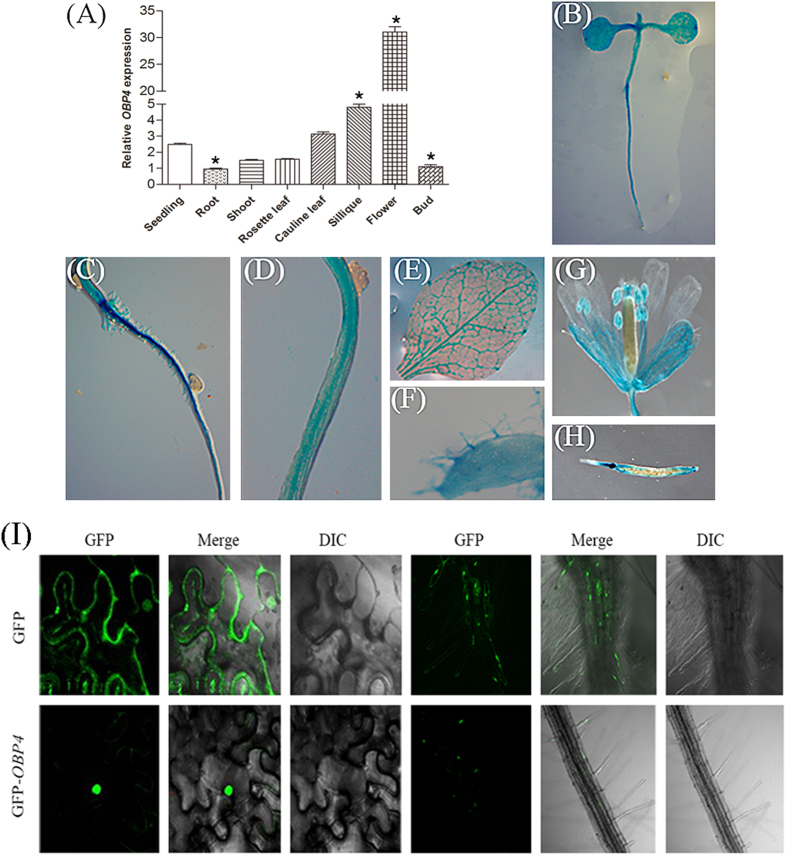
Tissue and cell cycle-dependent *OBP4* expression patterns and subcellular localization. (**A**) qPCR analysis of *OBP4* gene expression level. (**B**–**G**) GUS promoter analysis of *OBP4* tissue-specific expression. (**B**) Seedling at 6 days after germination. (**C**) Roots. (**D**) Hypocotyls. (**E**) Rosette leaves. (**F**) Trichome. (**G**) Sepal of flower and anther. (**H**) Siliques. (**I**) Subcellular localization of the vector control and *OBP4* in tobacco epidermal cells and transgenic *Arabidopsis* root cells. DIC, differential interference contrast, referring to bright-field images of the cells. Asterisks indicate significant differences, p < 0.05.

To investigate the expression pattern of OBP4 during development, qPCR was used to examine the OBP4 mRNA levels in different plant organs, including seedlings, roots, shoots, flowers, rosette leaves, cauline leaves, buds and siliques. Our results showed that OBP4 was ubiquitously expressed at different levels in all of the examined organs (Fig. 2A). Then, approximately 2.0 kb of the 5′-UTR promoter region of OBP4 was fused to the β-glucuronidase (GUS) reporter gene to investigate OBP4 expression at the tissue level. The GUS signal was observed in the silique, trichomes, flower organs and leaves (Fig. 2F–H). GUS was also detected in the seedlings, hypocotyls and roots (Fig. 2B–E). Furthermore, the subcellular localization of the OBP4 protein was analyzed. Figure 2I shows that the GFP control protein was observed in both the nuclei and the cytoplasm, whereas the 35 S::OBP4::GFP protein was localized in the nuclei when transiently expressed in tobacco epidermal cells. The same results were observed in transgenic roots stably expressing OBP4::GFP. These results indicate that OBP4 is a nuclear protein.

### Constitutive and inducible overexpression of *OBP4* alters cell proliferation and results in a dwarf phenotype

**Figure 3:**
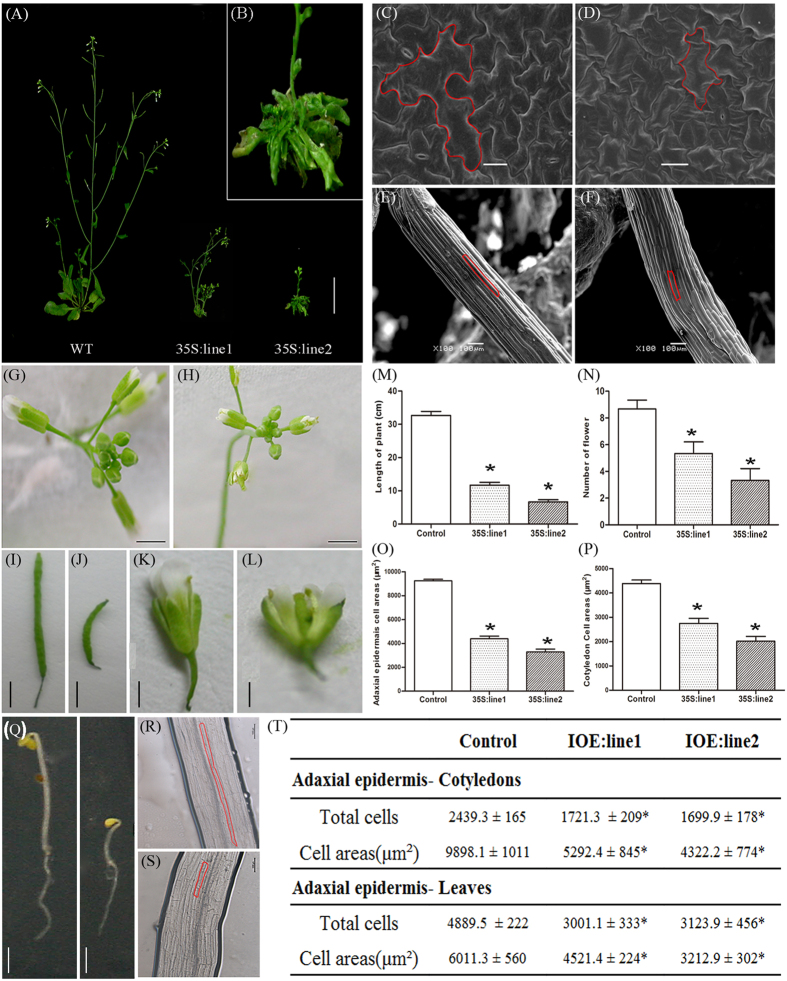
Phenotypes of transgenic plants overexpressing *OBP4.* Six-week-old (**A**) wild-type and (**B**) 35 S::*OBP4* plants grown under long-day conditions. The heights of the inflorescence stems of at least 7 plants were measured. (M) The plant height and (N) the flower number of the wild-type and 35 S::*OBP4* plants. Two-week-old adaxial epidermis of and basal part of hypocotyls from (**C,E**) wild-type and (**D,F**) 35 S::*OBP4* leaves were visualized by scanning electron microscopy. The red outlines indicate the cell boundary of one epidermal cell. Cell area analysis of (O) epidermal cells and (P) cotyledon cells. The bars represent the SD. Inflorescence morphology of (**G**) wild-type and (**H**) 35 S::*OBP4* plants. Silique analysis of (**I**) wild-type and (**J**) 35 S::*OBP4* plants. Flower morphology of (**K**) wild-type and (b) 35 S::*OBP4* plants. (**Q**) Seedlings of control (left) and pER8::OBP4 (right) plants grown on MS medium supplied with 20 μM estradiol in the dark. Hypocotyls of (**S**) control and (**R)** pER8::*OBP4* dark-grown seedlings (Scale bar = 100 μm). The red outlines indicate the cell boundary of one hypocotyl cell. (**T**) Analysis of epidermal cells of cotyledons and the fourth rosette leaf of wild-type and pER8::*OBP4* plants (data are the mean ± SD, n = 7). Scale bars = 5 cm in (**A**,**B**); 0.4 cm in (**K**,**L**); 0.3 cm in (**G–J**); 100 μm in (**C-F**). Asterisks indicate significant differences, p < 0.05.

To elucidate the mechanisms underlying the effects of *OBP4* on cell cycle progression and cell expansion, which influence plant development, we performed a phenotypic analysis of plants that were constitutively overexpressing *OBP4*. *OBP4* transgenic lines were screened and eight transgenic lines overexpressing *OBP4* were obtained, with the highest expression level approximately 32-fold to that of the WT at mRNA level (Fig. S2). We further attempted to increase the *OBP4* expression in transgenic *Arabidopsis* without success. Constitutive *OBP4* overexpression resulted in obvious defects in the plant development. Mature 35S::OBP4 plants had a greatly reduced plant size with small and curly rosette leaves (Fig. 3A,B). To investigate the reason for the arrested plant development, leaf cells and hypocotyl cells were counted and measured. In the one-week-old 35S::OBP4 seedling hypocotyls and the fourth leaves from three-week-old 35S::OBP4 plants, the cell numbers were decreased, and epidermal cell size was also reduced compared to that of the wild-type plants (Fig. 3C–F,O,P). The petals were shorter, the siliques produced were shorter and fewer, and seed setting did not occur (Fig. 3G–L). Furthermore, transgenic plants were characterized by shorter plant height and reduced flower number (Fig. 3M,N).

We also observed a similar seedling phenotype by inducing *OBP4* expression in pER8::*OBP4* transgenic plants. pER8::*OBP4* plants treated with 20 μM estradiol were smaller than WT and showed growth arrest 4 days after induction in dark (Fig. 3Q,R,S). At the cellular level, the hypocotyl cells of pER8::*OBP4* plants were smaller than those of WT (Fig. 3T). The situation was similar for the *35S::*OBP4 plants which were smaller than WT. Unfortunately, *OBP4* loss-of-function mutant was not available in the public seed collections and RNAi approaches were unsuccessful. To convert OBP4 into a transcriptional activator, we fused it with the strong activation domain VP16. The resulting *OBP4*::VP16 causes strong transcriptional activation. Therefore, we transformed pER8::*OBP4*::VP16 as a potential negative *OBP4* allele into plants. Further we obtained pER8::*OBP4*::VP16 transgenic plants, and after four days of estradiol induction, the leaves were obviously larger than those of the control (Fig. S4). These results demonstrated that OBP4 can regulate plant development.

### *OBP4* negatively regulates G2-to-M phase transition and promotes endocycle onset

**Figure 4:**
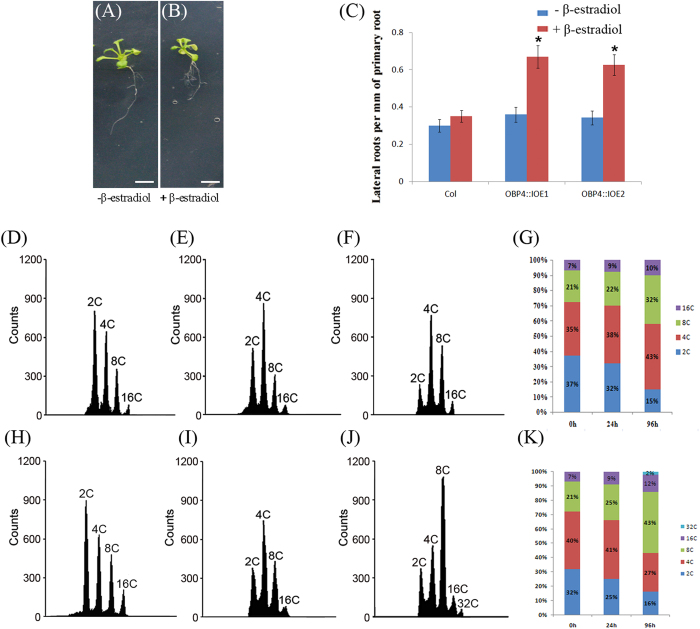
Flow cytometric analysis of cell cycle progression. (**A–C**) Plant phenotypes in two induced *OBP4* overexpression lines (pER8 line 1, pER8 line 2). The images are from the mid-portion of each root of 13-day-old plants grown on MS solid medium (**A**) without or **(B**) with 20 μm estradiol induction for 5 days. Scale bar = 0.5 cm. (**C**) The root lengths and lateral root number were measured before and after induction. Flow cytometric analysis of wild-type (**D–G**) and pER8::*OBP4* (**H–K**) in DAG 7, DAG 8, and DAG 11 seedlings after 0, 1, and 4 days of 20 μm estradiol induction, respectively. 2 C, 4 C, 8 C, 16 C and 32 C represent the DAPI signals that correspond to nuclei with different DNA contents. Flow cytometric analysis was repeated three times. The average ( ± SD) from more than 5 seedlings for each time point is presented. Asterisks indicate significant differences, p < 0.05.

We hypothesized that G2-to-M phase transition is defective in pER8::*OBP4* plants. To investigate the mechanism underlying *OBP4* regulation of G2-to-M phase transition, we determined whether *the transgenic* plant cells showed defects during cell cycle progression, particularly G2-to-M phase transition, such as G2 phase arrest^2^,^39^ and early endocycle entry^49^. Overexpression of *OBP4* affected cell proliferation in suspension cells (Fig. S5). To assess whether G2-to-M phase transition is defective in pER8::*OBP4* plants, we measured the DNA content in pER8::*OBP4* plant cells by flow cytometry. Before induction, at 7 days after germination (DAG), the ploidy level distribution was similar to that of wild-type (Fig. 4D,H); Nuclei isolated from both WT and pER8::*OBP4* seedlings displayed four primary peaks that corresponded to 2 C, 4 C, 8 C, and 16 C DNA content. After 1-day induction with estradiol, pER8::*OBP4* plants showed an increased proportion of 8 C cells and a decreased proportion of 2 C and 4 C cells (Fig. 4E,I). After 4-day induction, the proportion of 8 C cells greatly increased in the pER8::*OBP4* plants, and the proportion of 4 C and 2 C cells further decreased. Additionally, 32 C cells appeared in the β-estradiol-induced plants (Fig. 4F,J). These results suggest that more pER8::*OBP4* plant cells escaped from the cell cycle compared to control plants and that early endocycle onset occurred.

We further measured the DNA content in pER8::*OBP4*::VP16 transgenic plant cells by flow cytometry. Before induction, at 9 days after germination (DAG), the ploidy level distribution was similar to that of the control. After the plants were induced for 4 days, pER8::*OBP4*::VP16 transgenic plants had a decreased proportion of 8 C and 32 C cells. In contrast, the vector control and the wild-type plants showed similar DNA content before and after estradiol treatment (Fig. S4 D–G). Thus, Delayed endocycle onset was observed in the *OBP4*::VP16 plants.

### *OBP4* negatively regulates cell cycle genes and cell wall expansion factors

**Figure 5:**
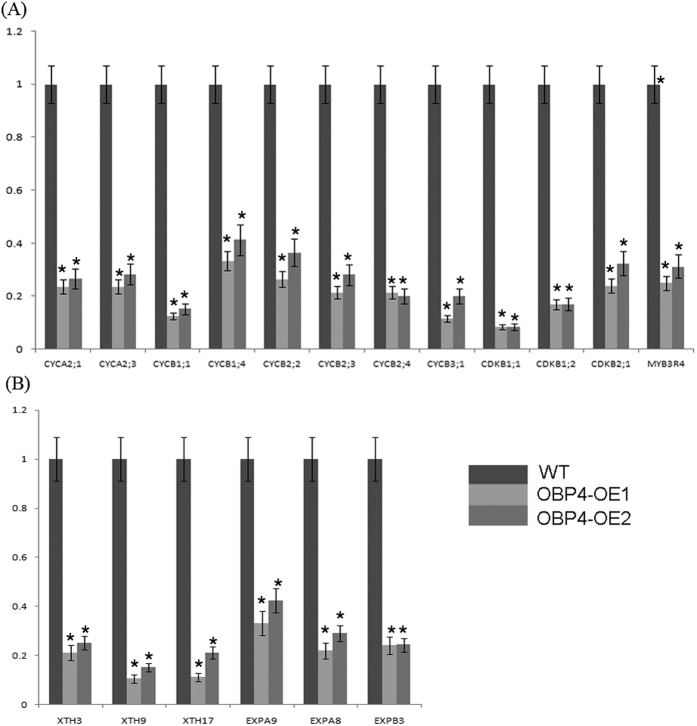
*OBP4* regulates the expression of core cell cycle genes and cell expansion factors. (**A**) Using qRT-PCR analysis, we confirmed that the cyclin genes CYCA2;1, CYCA2;4, CYCB1;1, CYCB1;4, CYCB2;2, CYCB2;3, CYCB2;4, CYCB3;1, CDKB1;1, CDKB1;2, CDKB2;1 and a G2-specific regulator, MYB3R4, were repressed strongly in *OBP4*-OE plants. (**B**) The cell expansion factor genes *XHT3*, *XTH9*, *XTH17*, *EXPA9*, *EXPA8* and *EXPB3* were strongly repressed in *OBP4*-OE. Expression levels were normalized to *ACTIN2* expression as an internal control. The values shown are the means ± SD. Experiments were performed three times. Asterisks indicate significant differences, p < 0.05.

Because the OBP4 protein is a nuclear-localized DOF transcription factor, we hypothesized that *OBP4* controls plant development by regulating gene expression. To verify this hypothesis, we investigated the ability of *OBP4* to regulate downstream target genes. We analyzed differentially expressed genes between wild-type plants and plants that constitutively overexpressed *OBP4*. We identified the genes encoding cell wall expansion factors, such as xyloglucan endo -transglucosylation genes (*XTH3*, *XTH9*, *XTH17*) and expansion genes (*EXPA8*, *EXPA9*, *EXPB3*), and cell cycle-related genes, such as cyclin genes (*CYCA2;1*, *CYCA2;3*, *CYCB1;1*, *CYCB2;1*, *CYCB2;3*, *CYCB3;1*,) cyclin-dependent kinase genes *CDKB1;1*, *CDKB1;2*, *CDKB2;1*) and cell cycle-related transcription factors (*MYB3R4*). These genes were markedly down-regulated in the 35 S::*OBP4* transgenic lines as determined by qPCR analysis (Fig. 5).

**Figure 6:**
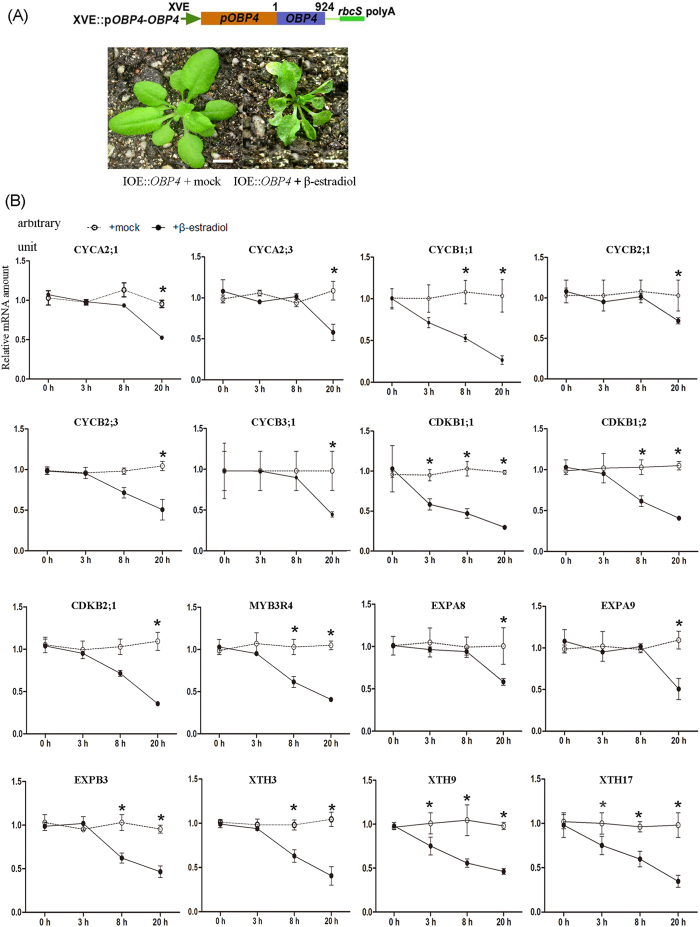
Search for immediate downstream genes of *OBP4* using an estradiol-inducible transgenic plant. (**A**) pER8::_Pro_OBP4::*OBP4* construct. The *OBP4* gene was driven by the *OBP4* promoter. The pER8 plant is shown before and after β-estradiol treatment. Leaf development was partly inhibited by estradiol induction in the seedlings. Scale bar = 0.5 cm. (**B**) Time-course induction analysis of the 10 cell cycle-related genes and 6 cell wall modification genes performed using real-time RT-PCR analysis. pER8::_Pro_OBP4::*OBP4* plant after a single addition of estradiol (20 μm). RNA samples were extracted from pER8::_Pro_OBP4::*OBP4* leaves at 0, 3, 8, or 20 h after mock (circles) or estradiol (dots) treatment. Vertical axis indicates the relative mRNA amount after β-estradiol treatment. Horizontal axis indicates the time after treatment. The bars indicate the SD. Asterisks indicate significant differences, p < 0.05.

We further identified genes that were immediately expressed upon activation of *OBP4*. The estradiol induction system is successful at inducing plant transcription factors^47^. We generated a homozygous transgenic plant (pER8::_Pro_OBP4*::OBP4*) with the *OBP4* promoter driving a fusion of *OBP4* cDNA under the β-estradiol-inducible promoter in the Col-0 background. The transgenic plant was similar to wild-type plants under normal conditions. In contrast, estradiol spray clearly inhibited leaf development; upward curling leaves were observed (Fig. 6A). Similar to stimulation of steroid receptors in animals, estradiol treatment led to transport of the *OBP4* protein from the cytoplasm to the nucleus, resulting in downstream events. Furthermore, we performed qPCR analysis using RNA samples within 20 h of induction. Figure 6B indicates that the 16 isolated genes were clearly down-regulated after estradiol treatment for 20 h. The large number of expression changes at 20 h after estradiol induction suggests that secondary targets of *OBP4* might exist. Genes with reduced expression were further examined for their induction kinetics. We profiled the selected genes at 3 h and 8 h after induction to identify direct targets. Among these genes, we identified 5 genes that were consistently down-regulated at 3 h and 8 h after induction: two core cell cycle proteins (CYCB1;1 *and* CDKB1;1*) and three cell wall expansion factors* (*XTH3*, *XTH9*, and *XTH17*). Moreover, their expression levels were higher in the OBP4*::VP16 transgenic* plants (Fig. S3). These results show that *CYCB1;1*, *CDKB1;1*, *XTH3*, *XTH9*, and *XTH17* are putative genes immediately downstream of *OBP4*.

**Figure 7:**
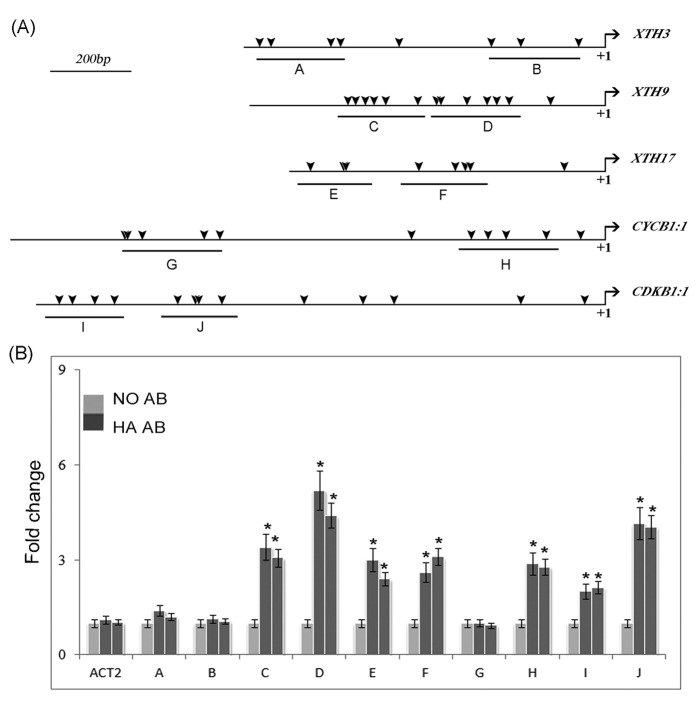
ChIP analysis to verify *OBP4* target genes. (**A**) The sequence regions used for ChIP assays are marked in the gene promoters (**A–K**, top panel). DOF binding motifs are indicated as arrowheads. (**B**) pER8::*OBP4*::HA-1 and pER8::*OBP4*::HA-2 transgenic plants grown on MS-agar plates for 2 weeks were used for ChIP assays (bottom panel). The enrichment shown was calculated as the DNA level of each fragment in the β-estradiol-treated sample divided by that in the DMSO-treated sample. Anti-HA antibody was used to precipitate *OBP4*-HA. Three measurements were averaged for individual assays. Bars indicate the SD. The values in Col-0 plants were set to 1 after normalization to ACT2 for qPCR analysis. Asterisks indicate significant differences, p < 0.05.

**Figure 8:**
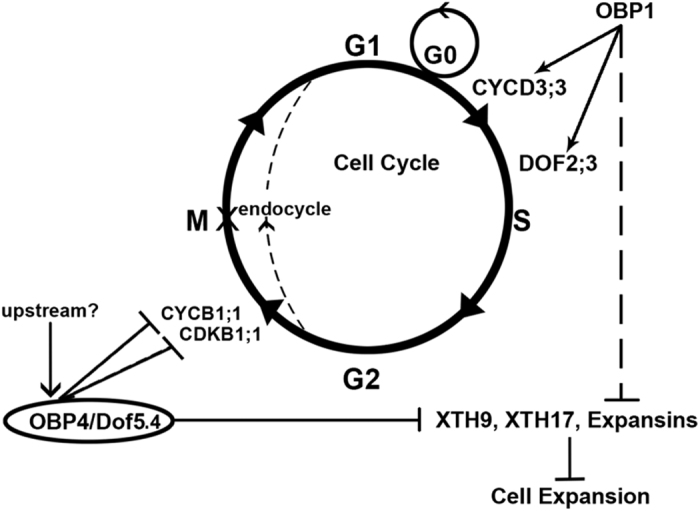
Model of *OBP4* function in cell cycle regulation and cell expansion. Model of the OBP4 and OBP1 regulatory network. We inferred that the cell cycle regulators *CYCB1;1* and *CDKB1;1* and the cell expansion factors *XTH9* and *XTH17* are *OBP4* target genes. We also showed that *OBP4* negatively affects both cell cycle progression and cell expansion. *OBP4* overexpression resulted in arrested development of *Arabidopsis* plants, with fewer and smaller cells. While *OBP1* positively regulates CYCD3;3 and *DOF2.3.* OBP1 negatively affects cell expansion. The dotted line indicates entry to endoreplication.

The OBP4 transcription factor belongs to the DOF subfamily, whose predicted DNA binding sites are [A/T]AAAG and CTTT[A/T]^57^,^58^,^59^. Based on these DNA sequences, we found many DOF binding sites in the promoter regions of the *CYCB1;1*, *CDKB1;1*, *XTH3*, *XTH9*, and *XTH17* genes (Fig. 7A). To verify whether OBP4 can bind to these consensus sequences in planta, we performed ChIP analysis to test the relative enrichment of OBP4 in the selected fragments. An antibody directed against HA was used to precipitate *OBP4*::HA and its crosslinked DNA, which was isolated from 2-week-old pER8::*OBP4*::HA seedlings after estradiol induction for 36 h, and pER8::HA seedlings without induction served as a negative control. Immunoprecipitated DNA crosslinked with *OBP4*::HA was analyzed by real-time PCR using the gene-specific primers listed in Supplemental Table 2. OBP4 protein enrichment was indeed detected at the promoter regions of the *CYCB1;1*, *CDKB1;1*, *XTH9*, and *XTH17* genes containing the predicted DOF binding sites (Fig. 7B). In sum, we conclude that *OBP4* can transcriptionally regulate both cell cycle progression and cell expansion by regulating key cell cycle genes and cell expansion factors (Fig. 8).

## Discussion

In most eukaryotic cells, cell cycle progression is an ordered series of events during which the chromosomes are duplicated and one copy of each duplicated chromosome segregates into each of the two daughter cells. Cell cycle regulation is critical for the normal development of multicellular organisms. The cell cycle is controlled by numerous mechanisms that ensure correct cell division, i.e., regulation of cyclin-dependent kinases (CDKs) by cyclins, CDK inhibitors and phosphorylation^50^. Fundamentally, the molecular mechanisms controlling the primary events in cell cycle progression are similar to those in eukaryotic cells. Thus, research on diverse organisms has contributed to the growing understanding of how these events are coordinated and controlled in plants^10^. However, the plant cell has a cell wall, and the mechanisms of plant cell proliferation that are coordinated with cell wall expansion and contribute to plant growth remain unclear. A number of DOF proteins have been reported to participate in plant development and to respond to environmental stimuli. Previous results have shown that *OBP1* regulates cell cycle re-entry by targeting *CYCD3;3* and *DOF2.3*^47^. Here, we report that another plant-specific OBP family protein, OBP4, is a negative regulator of cell division and plant growth. This study demonstrates the *OBP4*-mediated inhibition of plant growth via defective cell cycle progression and cell wall expansion.

### *OBP4* is a positive regulator of the endocycle during plant development

To characterize the function of *OBP4*, we examined transgenic plants with up-regulated or reduced *OBP4* activity. The 35S constitutive and inducible overexpression transgenic plants presented obvious growth defects (Fig. 3A,B). While overexpression of OBP4 fused to an activation domain promoted plant growth (Fig. S3). *OBP4* was expressed in all of the examined organs but at different levels (Fig. 2B–H). The *OBP4* expression data from qPCR analysis were consistent with the GUS staining results (Fig. 2A). The timing and localization of *OBP4* expression suggest that *OBP4* has a role in cell cycle regulation. In inducible *OBP4* transgenic plants, the cell size and other characteristics related to cell division were inhibited. *OBP4* overexpression caused defective G2-to-M phase transition.

Furthermore, we hypothesized that *OBP4* plays a role in the onset of the endocycle^35^. Indeed, *OBP4* activation resulted in a longer G2 phase and early endoreduplication onset (Fig. 4). These findings support the conclusion that endoreplication entry involves the engagement of B-type cyclins and cyclin-dependent kinases^19^. Cell proliferation was negatively controlled by *OBP4* activation, and subsequently, the rate of cell proliferation was lower than that of the control, eventually resulting in fewer and smaller cells. The expression pattern of *OBP4* together with its effect on cell cycle regulators, is a novel mechanism by which this protein controls cell proliferation.

### *OBP4* inhibits cell expansion by negatively regulating cell wall expansion factors

In contrast to animal cells, plant cells have cell walls. Cell morphogenesis involves the spatially controlled remodeling of the cell wall. Cell expansion is believed to be controlled by a balance between the drive for expansion and localized wall loosening^43^. There are two types of proteins that are involved in this process. Xyloglucan endotransglycosylases cleave the glycan backbone and transfer the cut end to an acceptor molecule. Expansins appear to loosen hydrogen bonds between cellulose microfibrils and cellulose fibers^11^,^26^. *XTH9* may be associated with cell expansion in *Arabidopsis*^18^. A T-DNA insertion mutant of *XTH17* showed low XET activity and had moderately shorter roots than wild-type plants^66^. Plants overexpressing *AtEXP10* have large organs with enlarged cells, supporting the role of expansion in cell enlargement. *Arabidopsis* has 26 expansin genes, and more than 5 expansin genes may act redundantly to influence plant growth^8^. To determine how *OBP4* affects cell expansion, we examined the expression levels of several characterized *Arabidopsis* genes involved in cell expansion. The expression levels of the expansion genes *AtEXPA3*, *AtEXPA5*, *AtEXPA8*, *AtEXPA9* and *AtEXPB3* were significantly lower in 35S::*OBP4* plants than in wild-type plants. *AtEXP3* has been proposed to promote cell expansion^24^, and the *atexp5* mutant has smaller leaves than wild-type^40^. These results suggest that reduced cell size in *OBP4* overexpression lines might result from decreased expression of particular expansion genes. *OBP4* may negatively regulate cell wall expansion factors to modify cell wall plasticity during the cell growth stage.

The well-accepted “compensated cell enlargement theory” indicates that a reduced cell number could be compensated by increased cell size and vice versa^50^. Constitutive and inducible activation of *OBP4* in plants resulted in a significant reduction in both cell size and cell number. The leaves of 35S::*OBP4* plants were characterized by fewer cells and a reduced cell size, which could not be explained by the compensation theory and which might suggest that *OBP4* targets not only cell proliferation but also cell expansion. In contrast, the reduced cell size correlated well with the reduced level of the endocycle (fewer cells entering the endocycle), which has often been reported previously. To identify possible molecular mechanisms that could explain the changes in cell size, we examined the expression of genes encoding growth-related proteins among transcripts that were down-regulated in our profiling data and identified a large number of genes encoding cell wall-loosening enzymes, which are essential for cell growth and expansion^30^. Therefore, *OBP4* can also affect growth by targeting the expression of genes encoding cell wall loosening enzymes. In conclusion, *OBP4*, as a DOF family transcription factor, can modify the onset of the endocycle and cell growth during plant development. This characterization of the function of *OBP4* extends our understanding of the molecular mechanisms underlying cell cycle progression and cell expansion.

## Additional Information

**How to cite this article**: Xu, P. *et al*. *AtDOF5.4/OBP4,* a Dof Transcription Factor Gene that Negatively Regulates Cell Cycle Progression and Cell Expansion in *Arabidopsis thaliana*. *Sci. Rep.* **6**, 27705; doi: 10.1038/srep27705 (2016).

## Supplementary Material

Supplementary Information
