# Supplementary material for: AtDOF5.4/OBP4, a DOF Transcription Factor Gene that Negatively Regulates Cell Cycle Progression and Cell Expansion in Arabidopsis thaliana
Source: Sci Rep. 2016 Jun 14;6:27705. doi: 10.1038/srep27705 (PMC4906354; doi:10.1038/srep27705)
Supplement: Supplementary Information [file srep27705-s1.doc]

## Supporting data

Article title: ***AtDOF5.4/OBP4,* a Dof Transcription Factor Gene that negatively Regulates Cell Cycle Progression and Cell Expansion in *Arabidopsis thaliana***

Authors: Peipei Xu^1^, Haiying Chen^1^, Lu Ying^1^ and Weiming Cai^1*^

**Supplementary material**

Supplementary data are available online.

**Supplementary** **Table S1.** Primers used in plasmid construction.

**Supplementary Table S2.** Gene-specific primers used in qPCR experiments.

**Supplementary** **Table. S3.** Cellular analysis of the pER8::*OBP4*::VP16 transgenic plants.

**Supplementary Fig. S1.** Homologous alignment of *OBP* proteins.

**Supplementary Fig. S2.** Analysis of the 35S::*OBP4* transgenic plants.

**Supplementary** **Fig. S3.** Generation and flow cytometry analysis of the VP16-OBP4 transgenic plants.

**Supplementary** **Fig. S4.** Variation of *OBP4* target gene expression in the pER8::*OBP4*::VP16 transgenic plants.

**Supplementary** **Fig. S5.** OBP4 overexpression affects cell proliferation in suspension cells.

**Supplementary** **Fig. S6.** Time-course induction analysis of OBP4 target genes in the presence of cyclohexamide.

**Supplementary Fig. S7.** Flow cytometric analysis of pER8::*OBP4-2* before and after estradiol induction.

**Supplementary Fig. S8.** Flow cytometric analysis of pER8::*OBP4* hypocotyl cells.

**Table S1 Primers used in plasmid construction.**

| Transgenes | Primers (Sequence 5’-3’) |
| --- | --- |
| pHB-*OBP4* | 5'- CAAGCTT ATGCAAGATATTCATGATTTCTCCATG-3′^a^ |
|  | 5'- GGTCTAGATCAAGGAAGGTAGAGACCACTCTGATC-′^a^ |
| pER8-*OBP4* | 5'- CCCTCGAG ATGCAAGATATTCATGAT-3′^a^ |
|  | 5'- GGACTAGT TCAAGGAAGGTAGAGACC-3′^a^ |
| SALK_118463C | 5'- ATTCCAAAAGAAGCTTCTCGC -3′ |
|  | 5'- CTGATGAGCTCTCATCCTTCG -3′ |
| *OBP4*-SRDX | 5'- CC AAGCTT ATGCAAGATATTCATGAT -3′ |
|  | 5'- GGATCCTTAAGCGAAGCCCAGACGAAGCTCAAGATCC  AGGTCAAG AGGAAGGTAGAGACCAC-3′ |
| VP16-*OBP4* | 5'- CC AAGCTT CTG GAT ATG GCC GAC TTC GAG TTT GAG CAG ATG TTT ACC GAT GCC CTT GGA ATT GAC GAG TAC GGT GGG ATGCAAGATATTCATGAT -3′ |
|  | 5'- GGATCC AGGAAGGTAGAGACCAC-3′ |
| *OBP4*::3'RNAi | 5'-CG GGATCC TCTAGATCCGAATATGGGATTTGAACCG-3′ |
|  | 5'- C GAGCTC CTGCAG ATTTGCACTCTTGGGATATT -3′ |
| double35S: :*OBP4*: :GFP | 5'-GGAATTC ATGCAAGATATTCATGATTTCTC-3′ |
|  | 5'- GGTCGACAGGAAGGTAGAGACCACTCTGA-3′ |
|  | 5’-GGTACCATGGTAGATCTGACTA-3’ ^b^ |
|  | 5’GAGCTCTCACACGTGGTGGTGGT-3’ ^b^ |
| OBP4-qPCR primer | 5'- TTTCCGGCGGTTTCAGCTAT -3′ |
|  | 5'- CTCAACCTGAGCCGTTCGAT -3′ |

Underlined are restriction digestion sites.

Sequence shown in bold is used to clone HA-tag.

a: primers used for clone OBP4 gene cDNA in *Arabidopsis*. b: specific primers for GFP.

**Table S2 Gene-specific primers used in qPCR experiments.**

| Genes | | Primers (Sequence 5’-3’) |  |
| --- | --- | --- | --- |
| *CYCA2;1* | | 5'-ACGTTTCTCAGGCGGTTCAT-3’ |  |
|  |  | 5'-TGGTCGAGTGTCCATCTTGC-3’ |  |
| *CYCA 3;1* | | 5'-CATTCTTTCAAATCACCCGC-3’ |  |
|  |  | 5'-CTAGGTTCATCATCCGTCCA-3’ |  |
| *CYCB 1;1* | | 5'-GCTTCTGCAATCTACGCAGC-3’ |  |
|  |  | 5'-CCAACAGCTTTGCACAGTCC-3’ |  |
| *CYCB 2;1* | | 5'-CGAGAAGATGAGAGCAATAC-3’ |  |
|  |  | 5'-TCGATGAGCCAGTCAATAAG-3’ |  |
| *CYCD 3;1* | | 5'-GCAAGTTGATCCCTTTGACC-3’ |  |
|  |  | 5'-CAGCTTGGACTGTTCAACGA-3’ |  |
| *CDKB 1;1* | | 5'-TGAGATGGTTCGGAGGCAAG-3’ |  |
|  |  | 5'-AGTCACGCAGTGTGGAAACA-3’ |  |
| *CDKB 2;1* | | 5'-CACGTCGTCAGGTTAATGGA-3’ |  |
|  |  | 5'-TGTTCTTGCCAGTGCTACGG-3’ |  |
| *CYCA1;1* | | 5'-CTTCGTGCTTCTGAGGCTAA-3’ |  |
|  |  | 5'-CTTCAGACACCTCAATAAGCC-3’ |  |
| *CYCA2;1* | | 5'- CCGCGAAATAGTTGGCTAAG-3’ |  |
|  |  | 5'-CGTTTCTCAGGCGGTTCATT-3’ |  |
| *CDKB1;1* | | 5'-CCTCGCAGCTGTGGAATATG-3’ |  |
|  |  | 5'-CCTCCATTCACTCTCAACAG-3’ |  |
| *CYCB1;1* | | 5'-ATTTGGGTCTTGGTCGTGCTT-3’ |  |
|  |  | 5'-AAAGAGCTTGCCTCCGAACC-3’ |  |
| *CDKB1;2* | | 5'-AGGAGGCAAGCTCTTTTCCC-3’ |  |
|  |  | 5'-GATAAGTCTTGCGGCTCCCA-3’ |  |
| *AT5G11510* | | 5'-CTGATCCAAAAGGCCAGACGACAT-3’ |  |
|  |  | 5'-TGCCTGGAAGGTCGGATAATGGAA-3’ |  |
| *CDKB1;1* | | 5′-ATGGAGAAGTACGAGAAGCT-3′ |  |
|  |  | 5′-TCAGAACTGAGACTTGTCAA-3′; |  |
| *Actin*7 | | 5′-GATGCTTACGTTGGTGATGA-3′ |  |
|  |  | 5′-CTGACTCATCGTACTCACT-3′ |  |
| Genes | Primers (Sequence 5’-3’) | | |
| *XTH3* | TCTGGAACGGTGACGATTGG | | |
|  | TGCCTGCATTCCACCAGTAG | | |
| *XTH9* | TGTCAACGAAGGCGAAGTCA | | |
|  | GGACTATGTAAGGCTCGCCGG | | |
| *XTH17* | CGCTCGACAAATCCTCTGGA | | |
|  | CTCATCCCACGTAGTTCCCG | | |
| *ATEXPB3* | CCCAGTCATTTATCGCCGGA | | |
|  | CTTGGCGAATGTGCATGGAG | | |
| *ATEXPA6* | ACATAGTGCGGCTAGGTGTG | | |
|  | CGGTCACTGCTTGTGACTCT | | |
| *ATEXPA3* | ACAGCCAAGGATACGGTGTG | | |
|  | CTCGGCTGAGCAAAATTCGG | | |
| *ATEXPA2* | GTGCATCCCTGGTTCCATCA | | |
|  | GACAGGAACGATCCCAGCTC | | |
| *ATEXPA15* | ATGGCTATGACGCTGGATGG | | |
|  | TAGAGGTTCCCGTAGCCACA | | |
| *ACTIN2* | *5’-TTCCCGTTCTGCGGTAGTGG-3’* | | |
|  | *5’-CCGGTATTGTGCTCGATTCTG-3’* | | |
| A | F：CTTCACTGAGCCGCACTTTGT | | |
|  | R：CAACACAAGATTTATGTATCT | | |
| B | F：TGTATAGAGAATTTGATGTGAC | | |
|  | R：TTGATTTAATTTTTGTTGTAAG | | |
| C | F：ACTATTAGATGCTTGTATCAAT | | |
|  | R：TGAGATGACGCCAAATTACAA | | |
| D | F：TGTAAAATGGTCCAATCTCTCT | | |
|  | R：CCATCTTCATAAAAAATCACTC | | |
| E | F: TTACATTTTAAGAAAAATGA | | |
|  | R: TTAAGCACCTATAAAACTAATTA | | |
| F | F: CACCACATTTTATCACTTATAAC | | |
|  | R: GATGGAGCCCATATATCGCTAT | | |
| G | F：GCAATGCTGAGATTTCCAAGGA | | |
|  | R：AGCTATGGTTCCCACACAATTG | | |
| H | F：ATCTAATTCACACAAATCTTTT | | |
|  | R：TAAAGAAAATAAATGAACCCCA | | |
| I | F：AAAAAGTTAGCTGTTGAAGTAA | | |
|  | R：TTGTTAATAATTCAGATGCCAG | | |
| J | F：AGTTGAGGACGAACATTCCCAA | | |
|  | R：TTCCATTCTTGCTTTGAGGAGT | | |

**
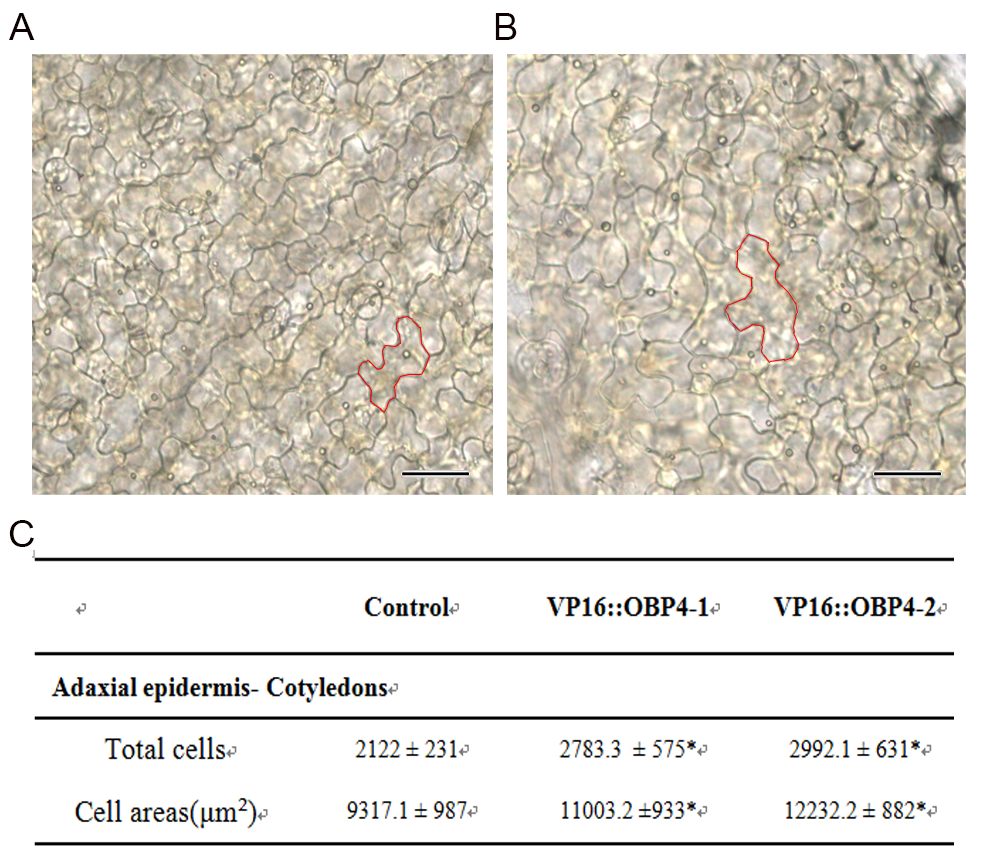
**

**Table S3. Cellular analysis of pER8::VP16::OBP4 transgenic plants.**

(A-B) Plant phenotypes in two *OBP4* induced activation lines (VP16::OBP4-1, VP16::OBP4-2). The images are from the mid-portion of each root of 9-day-old plants grown on MS solid medium (A) without or (B) with 20 μm estradiol induction for 4 days. (C) Analysis of epidermal cells of cotyledons in wild-type and VP16::*OBP4* transgenic plants after estradiol induction for 4 days. Asterisks indicate significant differences, p < 0.05. Scale bars = 100 μm cm in (A) and (B).


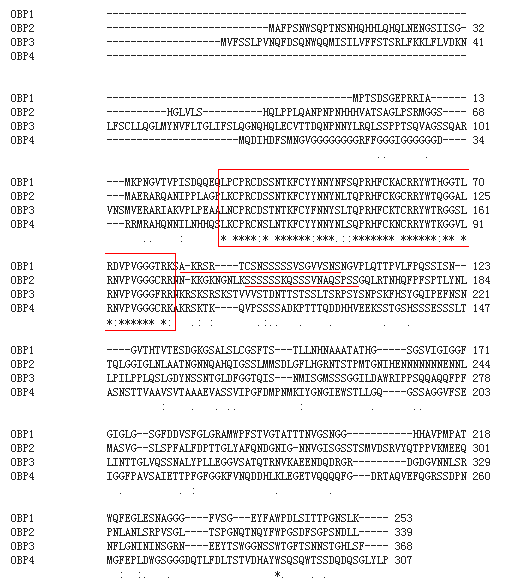


**Fig. S1** **Homologous alignment of *OBP* proteins**

The amino acid sequence for the open reading frame for each OBP proteins. The sequences are aligned with respect to the Dof domain of each OBP proteins. The Dof domain is boxed, and amino acids conserved among all the OBPs in the Dof domain are indicated by an asterisk. Serine-rich domains in OBP1 and OBP2 are under solid line. An asparagine-rich domain in OBP2 is shown under dotted line.


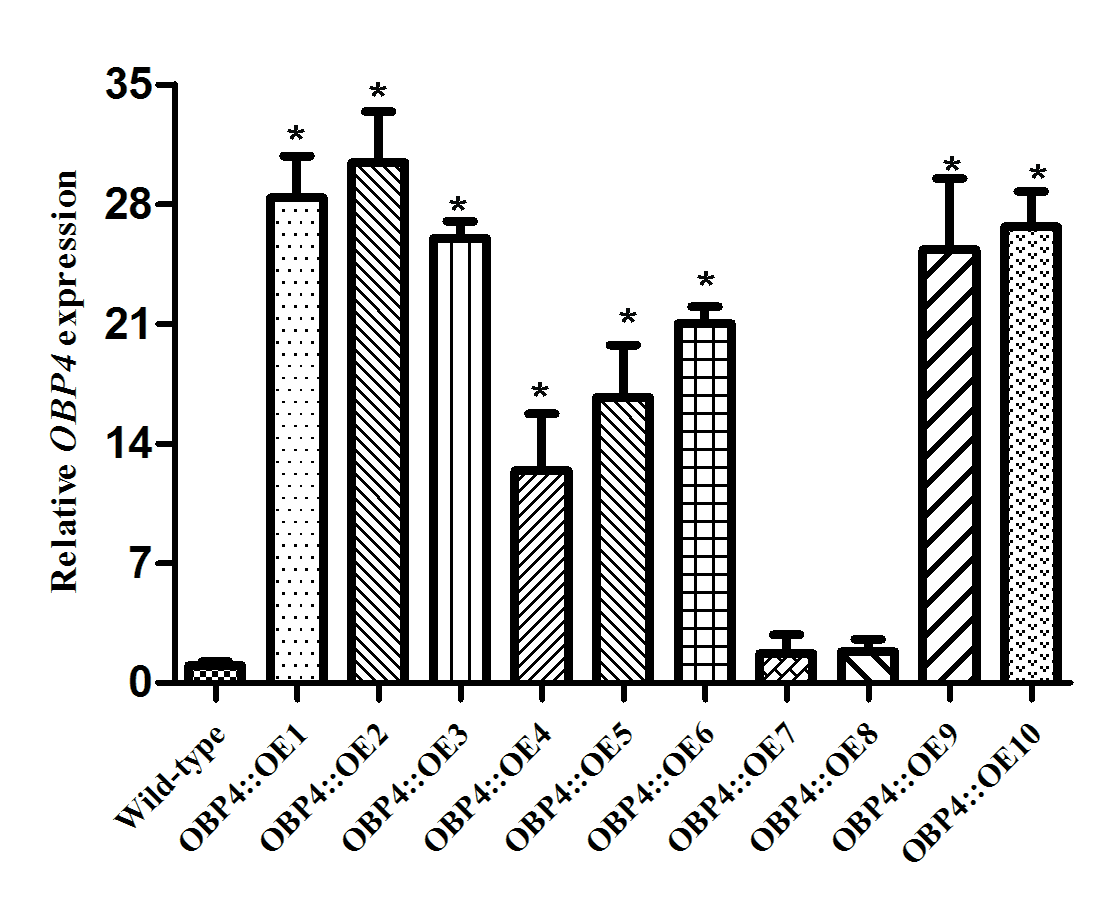


**Fig. S2** **Analysis of the 35S::*OBP4* transgenic plants** *OBP4* expression level in the 35S::OBP4 transgenic plants.


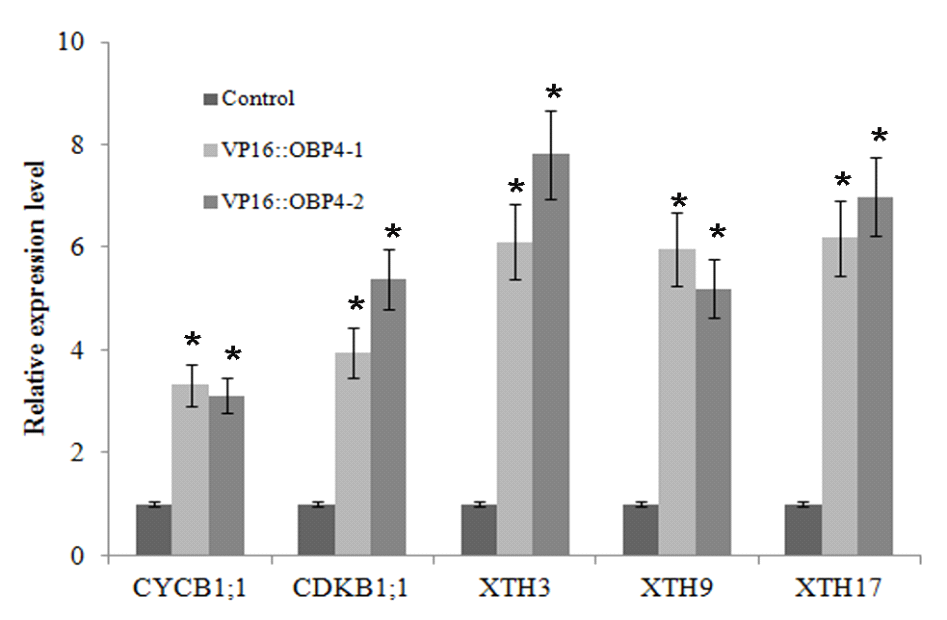


**Fig. S3**  **Variation of cell cycle and cell expansion related genes expression in the *OBP4*::SRDX transgenic plants after 20μm estradiol treatment for 3 days.**

**
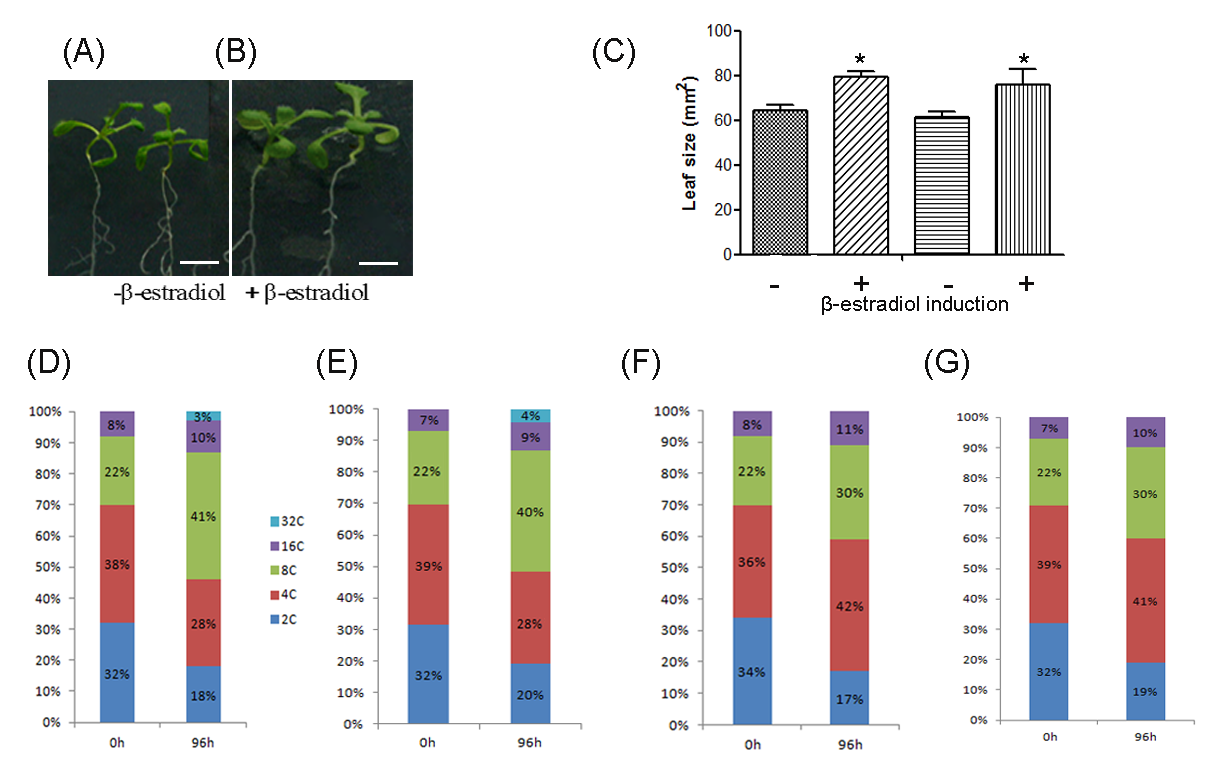
**

**Fig. S4. Generation and flow cytometry analysis pER8**::**VP16**::***OBP4* transgenic plants**

(A-B) Plant phenotypes in two *OBP4* induced activation lines (VP16::*OBP4*-1, VP16::*OBP4*-2). The images are from the mid-portion of each root of 13-day-old plants grown on MS solid medium (A) without or (B) with 20 μm estradiol induction for 4 days. Bar = 0.5 cm. (C) Leaf size were measured before and after induction. Flow cytometry analysis of wild-type (D) Vector control (E) and VP16::OBP4-1(F) VP16::OBP4-2(G) in DAG 9 and DAG 13 seedlings after 0 and 4 days of 20 μm estradiol induction, respectively. 2C, 4C, 8C, 16C and 32C represent the DAPI signals that correspond to nuclei with different DNA contents. Flow cytometry analysis was performed three times. Asterisks indicate significant differences, p < 0.05.

**
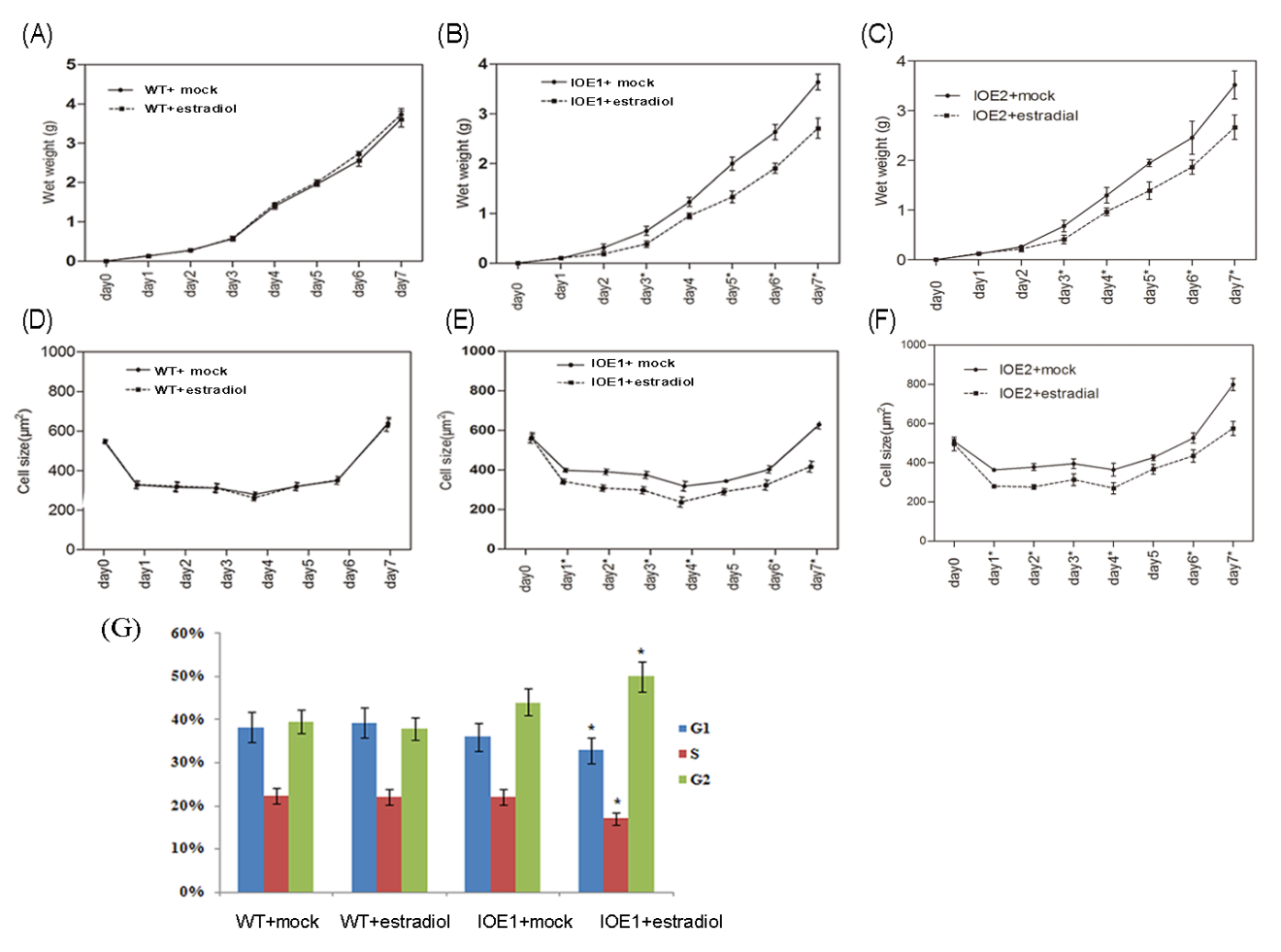
**

**Fig. S5. *OBP4* overexpression affect cell proliferation in *Arabidopsis* suspension cells**

Early stationary phase cells of wild-type and IOE::*OBP4* transgenic lines (IOE#1 and IOE#2) were transferred into fresh medium and sampled each day. For induction experiments a freshly prepared 200 mL culture was split into two 100 mL cultures, with one estradiol-induced and the other mock-induced. (A, B，C) Wet weight and (D, E，F) cell size were measured. Cell cultures lines were induced 7 days after subculture. (G) Phase distribution of nuclei measured by flow cytometry before and after estradiol induction. Asterisks indicate values that are significantly different from the respective controls (*P*< 0.05).

**
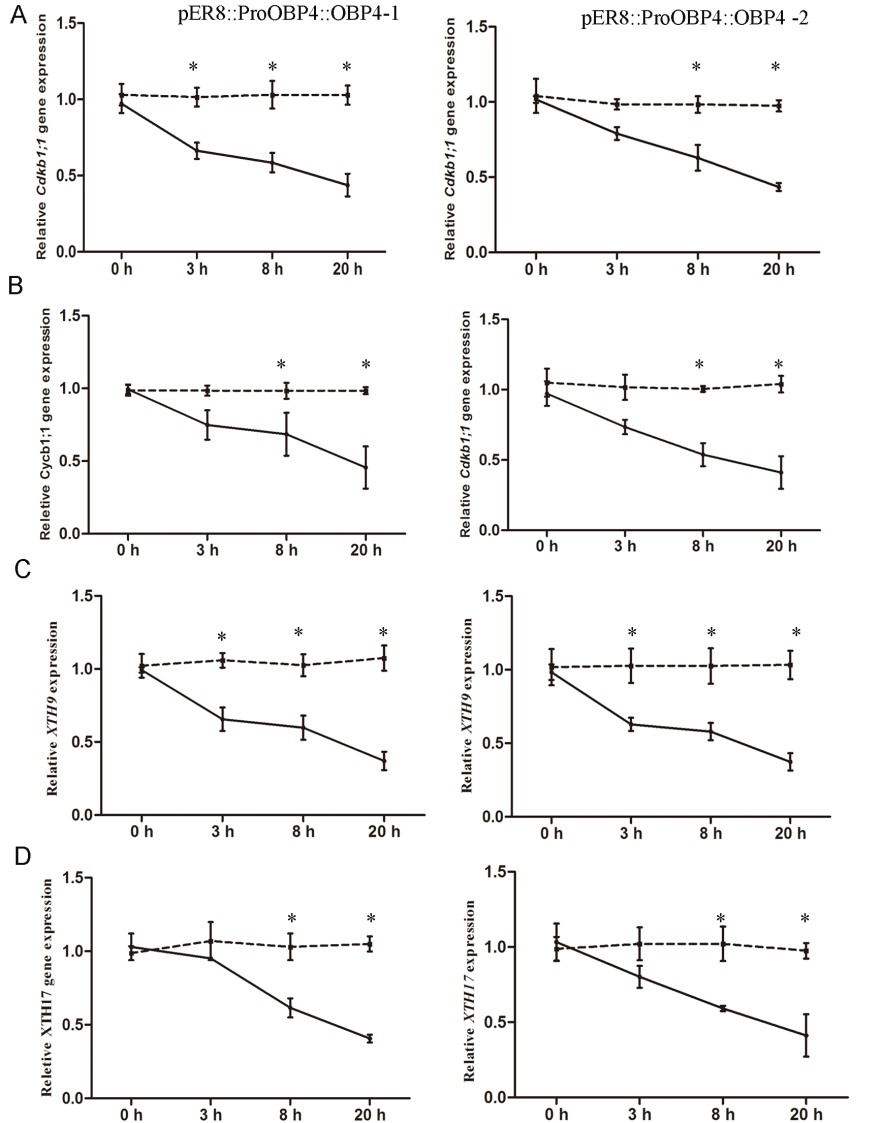
**

**Fig. S6. Time-course induction analysis of OBP4 target genes in the presence of cyclohexamide.**

RNA samples were extracted from pER8::_Pro_OBP4::*OBP4*-1 and pER8::_Pro_OBP4::*OBP4*-2 leaves at 0, 3, 8, or 20 h after mock or (20 μm) estradiol and cyclohexamide treatment. Vertical axis indicates the relative mRNA amount after treatment. Horizontal axis indicates the time after treatment. The bars indicate the SD.


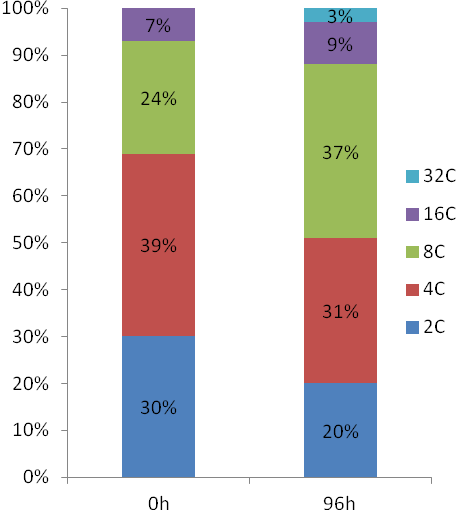


**Fig. S7. Flow cytometric analysis of pER8::*OBP4-2* before and after estradiol induction.**

Flow cytometric analysis of pER8::*OBP4*-2 in DAG 7 and DAG 11 seedlings after 0 and 4 days of 20 μm estradiol induction, respectively. 2C, 4C, 8C, 16C and 32C represent the DAPI signals that correspond to nuclei with different DNA contents. Flow cytometric analysis was repeated three times. The average (±SD) from more than 5 seedlings for each time point is presented.


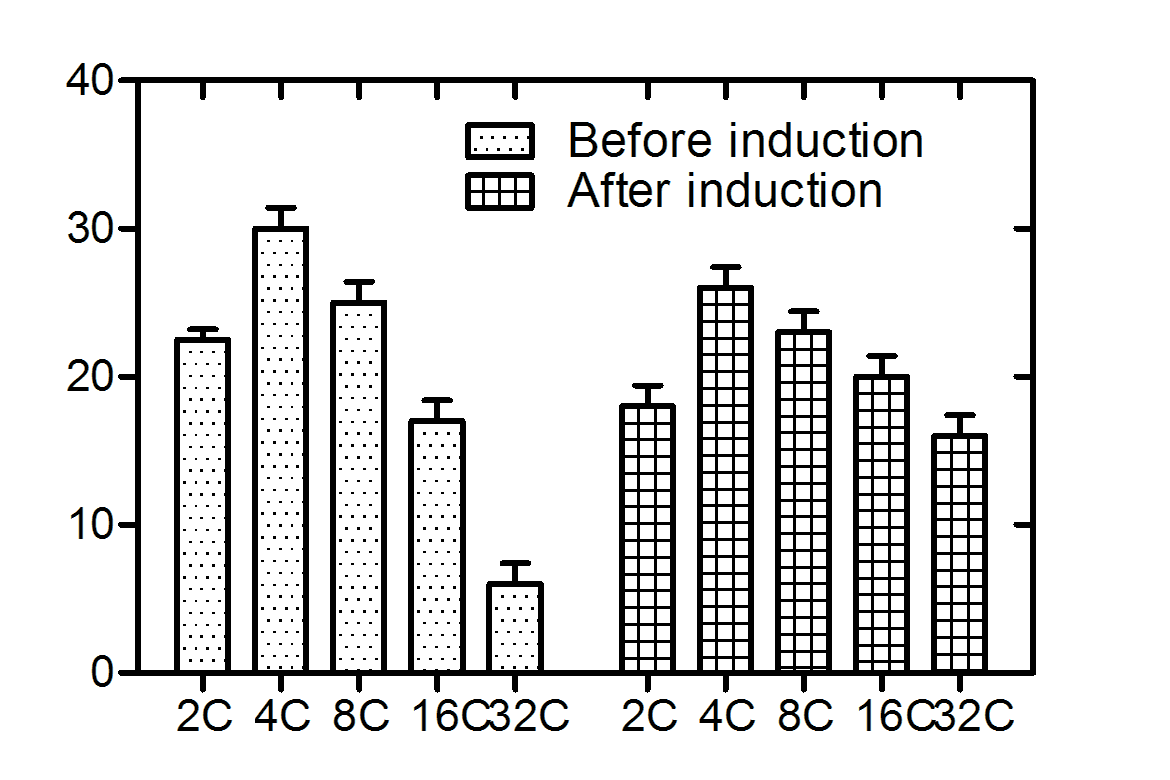


**Fig. S8. Flow cytometric analysis of pER8::*OBP4* hypocotyl cells.**

Flow cytometric analysis of wild-type and pER8::*OBP4* seedlings before and after 4 days 20 μm estradiol induction in hypocotyls. 2C, 4C, 8C, 16C and 32C represent the DAPI signals that correspond to nuclei with different DNA contents. Flow cytometric analysis was repeated three times.
